# The Role of the Immune System in Obesity and Insulin Resistance

**DOI:** 10.1155/2013/616193

**Published:** 2013-03-21

**Authors:** Payal S. Patel, Eric D. Buras, Ashok Balasubramanyam

**Affiliations:** ^1^Diabetes Research Center, Division of Diabetes, Endocrinology and Metabolism, Baylor College of Medicine, Houston, TX 77030, USA; ^2^Endocrine Service, Ben Taub General Hospital, Houston, TX 77030, USA

## Abstract

The innate immune system provides organisms with rapid and well-coordinated protection from foreign pathogens. However, under certain conditions of metabolic dysfunction, components of the innate immune system may be activated in the absence of external pathogens, leading to pathologic consequences. Indeed, there appears to be an intimate relationship between metabolic diseases and immune dysfunction; for example, macrophages are prime players in the initiation of a chronic inflammatory state in obesity which leads to insulin resistance. In response to increases in free fatty acid release from obese adipose depots, M1-polarized macrophages infiltrate adipose tissues. These M1 macrophages trigger inflammatory signaling and stress responses within cells that signal through JNK or IKK**β** pathways, leading to insulin resistance. If overnutrition persists, mechanisms that counteract inflammation (such as M2 macrophages and PPAR signaling) are suppressed, and the inflammation becomes chronic. Although macrophages are a principal constituent of obese adipose tissue inflammation, other components of the immune system such as lymphocytes and mast cells also contribute to the inflammatory cascade. Thus it is not merely an increased mass of adipose tissue that directly leads to attenuation of insulin action, but rather adipose tissue inflammation activated by the immune system in obese individuals that leads to insulin resistance.

## 1. Introduction

The obesity epidemic in the USA continues to expand at an alarming rate, with a 75% increase in prevalence since 1980 [1]. The Centers for Disease Control and Prevention (CDC) reports that more than one-third of USA adults and over one-sixth of children and adolescents are obese. The frequencies of other metabolic disorders have increased *pari passu*, including dyslipidemia, nonalcoholic steatohepatitis, and type 2 diabetes. Dysfunctional adipose tissue is central to all these conditions. Adipose tissue is increasingly recognized as a complex endocrine organ and not merely a depot for storage of fat. Adipose tissue in obese persons develops an inflammatory milieu which ultimately leads to insulin resistance. Although many components of the immune system have been found to play a role in either promoting or attenuating adipose tissue inflammation, macrophages are key players. This paper discusses the various stimuli and networks that lead to insulin resistance, with a primary focus on the role of macrophages in adipose tissue inflammation.

## 2. Macrophage Accumulation in Visceral Adipose Tissue in Obesity

Adipose tissue comprises not only adipocytes but also a heterogenous constellation of adipocyte precursors, nerve terminals, blood vessels, and leukocytes collectively termed the “stromal vascular compartment” (SVC). In 2003, pioneering studies by Xu et al. [2] and Weisberg et al. [3] demonstrated that obesity is associated with significant increases in macrophage number within the SVC of visceral adipose tissue (VAT). Weisberg et al. discovered the increase in macrophage number through gene expression profiling of VAT from multiple obese mouse models and lean controls. They found that levels of roughly 1300 genes directly correlated with body mass, and of the 100 most significantly correlated genes, 30% were macrophage-related [3]. Detailed analysis of SVC via flow cytometry revealed that macrophages make up approximately 40% of SVC cells from obese rodents, compared to only 10% of SVC cells in lean litter mates [3].

To understand the role of macrophages in adipose tissue insulin resistance, it is important to distinguish visceral fat from subcutaneous fat. Visceral fat is found in association with internal organs such as omentum, mesentery, and perinephric adipose tissue [4]. Visceral adiposity is predictive of hepatic steatosis, cardiovascular disease, and type 2 diabetes, whereas an increase in the mass of subcutaneous fat appears to pose little or no risk of these conditions [4]. Visceral and subcutaneous fat also differ in immune cell composition, particularly macrophages [5–7]. Similar to mouse models, increased macrophage accumulation has been demonstrated in the adipose tissue of obese humans and those with type 2 diabetes, with significantly more macrophages residing in visceral omental fat depot compared to subcutaneous inguinal depot in these subjects [5, 6]. Additionally, while macrophage content increases in both visceral and subcutaneous fat depot following a high-fat diet (HFD) feeding, the increase is severalfold greater in VAT [4]. Overall these findings indicate that increased macrophage accumulation in VAT may be a key pathologic feature of obesity and thereby of associated conditions such as type 2 diabetes, cardiovascular disease, and fatty liver.

## 3. Lipolysis, Resulting in an Increase of Free Fatty Acids, Promotes Adipose Tissue Macrophage Accumulation

What is the underlying factor that promotes the accumulation of adipose tissue macrophages (ATMs)? Kosteli et al. proposed that this phenomenon may be driven by alterations in adipose metabolic function and substrate fluxes, specifically increased concentrations of free fatty acids (FFAs) [8]. They suggested that obesity causes an increase in basal lipolysis, and the resultant increase in local extracellular free fatty acid concentrations could provide a chemotactic stimulus for entry and accumulation of macrophages. Basal lipolysis is chronically elevated in adipose tissue of obese compared with lean persons and in intra-abdominal compared with subcutaneous adipose tissue [8]. Consistent with this hypothesis, visceral adipose tissue depots contain more ATMs than abdominal subcutaneous depots [8]. Many details of this process remain to be clarified. Basal lipolysis could release a number of potential signaling molecules that could play a role in macrophage chemotaxis, for example, arachidonic acid products [8]. An additional intriguing feature is that lipolysis also increases during weight loss, and ATMs recruited in response to weight loss have a more anti-inflammatory phenotype (see below) than those in the adipose tissue of stably obese animals [8]. These differences suggest that additional factors are required for the entering macrophages to take on a proinflammatory phenotype.

## 4. Recruitment of Macrophages in Adipose Tissue

Recruitment of macrophages into adipose tissue is an early event in obesity-induced adipose depot inflammation. However, it is only one of several early events—overnutrition also causes adipocytes to release chemokines, such as monocyte chemoattractant protein-1 (MCP-1), providing a chemotactic gradient that attracts monocytes into adipose tissue [9]; these presumably can transform into tissue resident macrophages, that is, ATMs. A variety of chemokines released from ATMs can then recruit additional monocytes/macrophages, promoting a feed-forward process [9]. For instance, MCP-1, secreted primarily by macrophages and endothelial cells, is also secreted by adipocytes [10]. The MCP-1 ligand has high affinity for C-C motif receptor 2 (CCR2) on macrophages, and signals generated by this pathway stimulate macrophage migration into inflamed or damaged tissues [10, 11]. CCR2(−/−) mice display reduced ATM content, reduced proinflammatory cytokines, and improved systemic insulin sensitivity relative to body weight-matched wild-type (WT) controls [11]. There were some confounding phenotypic changes in the CCRT(−/−) mice; however Oh et al. showed that transplanting monocytes from the CCR2(−/−) mice into WT mice or WT monocytes into MCP-1(−/−) mice also resulted in decreased ATM accumulation [12]. Similar effects were observed when obese wild-type mice were treated with a CCR2 antagonist for a short term [11]. Conversely, transgenic mice overexpressing MCP-1 in adipocytes exhibited increased ATM levels, hepatic steatosis, and insulin resistance [13].

Another critical mediator of ATM accumulation is *α*4 integrin, which permits macrophage adhesion to endothelial cells and their subsequent transmigration through the endothelial barrier. Mice carrying a loss-of-function *α*4 integrin point mutation had reduced monocyte infiltration into VWAT and were protected from obesity-induced insulin resistance [14]. Cbl-associated protein (CAP), a known regulator of glucose transport (GLUT4) in adipocytes, also promotes macrophage mobility [15]. When the CAP gene is deleted from macrophages *in vivo*, the knock-out (KO) mice show a decrease in ATM content with reduced tissue inflammatory markers and cytokine concentrations [16]. The chemokine LTB4 is a potent chemoattractant for neutrophils—it is mainly produced by leukocytes, but is also expressed by adipocytes, and it augments MCP-1 expression in human monocytes, thus contributing to ATM infiltration [17]. Recent studies by Spite et al. have shown that mice lacking the gene encoding the LTB4 receptor, BLT1, manifested decreased inflammation in adipose tissue and liver and were protected from systemic glucose and insulin intolerance compared to WT littermates [18]. In keeping with these findings, deletion of other monocyte chemokine receptors such as CCR5 also mitigates ATM accumulation in HFD-fed mice. CCR5 and its ligands were robustly upregulated in VWAT of HFD-induced and genetically induced obese mice [19]. However, CCR5(−/−) mice were protected from insulin resistance, glucose intolerance, and hepatic steatosis induced by HFD feeding [19]. Taken together, these findings indicate that multiple chemoattractants draw macrophages, monocytes, and neutrophils into adipose tissue. An intriguing, unstudied possibility is whether these multiple chemoattractant-receptor pairs recruit different populations of macrophages or immune cells into adipose tissues.

## 5. Heterogeneity of Adipose Tissue Macrophages

**Figure 1:**
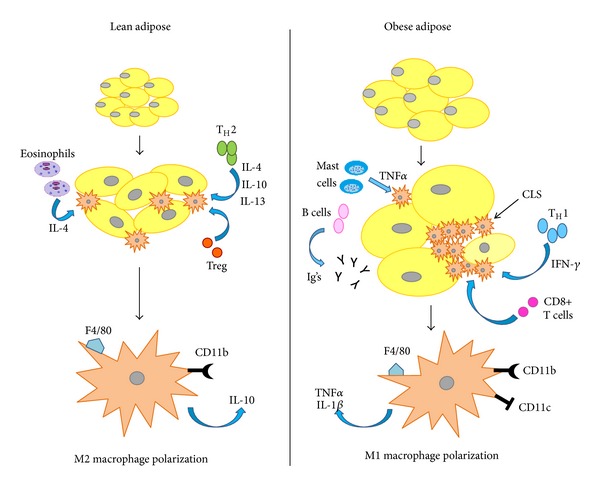
Role of the immune system in lean versus obese adipose tissue. In lean adipose tissue, T-helper type 2 (T_H_2) cells produce anti-inflammatory cytokines such as interleukin (IL)-4, 10, and 13 which promote alternatively activated M2 macrophage polarization. M2 polarization is also induced by regulatory T cells (Tregs) and eosinophils via IL-4. M2 macrophages secrete other anti-inflammatory signals such as IL-10 which maintain insulin sensitivity within lean adipose tissue. Conversely, T_H_1 type cytokines such as interferon (IFN)-*γ* stimulate M1 macrophage polarization in obese adipose tissue. Other immune cells are also increased in obese adipose tissue which contribute to insulin resistance including mast cells, B cells, and immunoglobulins (Igs). CD8(+) T cells promote ATM accumulation and proinflammatory gene expression and are also increased as well. Macrophages are not homogenously distributed throughout obese adipose tissue but rather aggregated around dead adipocytes forming crown-like structures (CLS). M1 macrophages are proinflammatory, secreting cytokines such as TNF-*α* and IL-1*β*. Macrophages are bone-marrow-derived myeloid cells hence both M1 and M2 macrophages express the myeloid cell surface markers F4/80 and CD11b. However, only the M1 population expresses the marker CD11c.

ATMs that reside in lean adipose tissue differ from those in obese adipose tissue. Classically activated macrophages (CAMs), termed M1, are generally stimulated by T-helper-1-type cytokines such as IFN-*γ* or bacterial by-products. M1 macrophages are proinflammatory, secreting cytokines such as TNF-*α* and IL-1*β*, and have high phagocytic and bactericidal potential [20]. M1 cells are generally recruited from the circulation in a CCR2-dependent manner, so it is likely that their accumulation in adipose tissues is possibly due to increased entry from the circulation. In contrast, T-helper-2-type cytokines such as interleukins (IL)-4, 10, and 13 promote alternatively activated macrophages termed M2. M2 macrophages have antiparasitic functions, secrete anti-inflammatory cytokines such as IL-10, and function in tissue repair and remodeling [21]. M2 cells are thought to be derived from replication of ATM resident macrophages—hence it is possible, but unproven, that their increase in adipose tissue is due to accelerated local multiplication (Figure 1).

Tissue macrophages respond to alterations in the local environment by changing their polarization status. Obesity not only promotes infiltration and migration of macrophages, but also induces a shift in macrophage balance towards the M1 phenotype [22]. In fact, obesity shifts the adipose M2 : M1 ratio from 4 : 1 in normal mice to 1.2 : 1 [23]. This shift is evident in the expression level of CD11c on ATMs. Macrophages are bone-marrow-derived myeloid cells; hence both M1 and M2 macrophages express the myeloid cell surface markers F4/80 and CD11b. However, only the M1 population expresses the marker CD11c, whereas M2 macrophages are CD11c(−) [24, 25]. M1, CD11c(+) recruited macrophages account for the majority of the increase in ATMs observed in obese adipose tissue, where >90% of recruited monocytes become CD11c(+) ATMs [24, 25]. Also, M1 correlates with insulin resistance as demonstrated by Patsouris et al. [26]. These investigators deleted CD11c(+) macrophages in mice using a genetic system in which the primate diphtheria toxin receptor (DTR) gene is driven by the CD11c promoter; the intention was to make CD11c(+) cells expressing DTR on their surface undergo apoptotic death when the animal was exposed to diphtheria toxin. In mice fed HFD for 16 weeks, diphtheria toxin exposure ablated about half of all ATMs, while also reducing myeloid cell content in the liver and skeletal muscle. Remarkably, only 24–48 hours after diphtheria toxin administration, glucose tolerance tests completely normalized in these mice, associated with improved insulin sensitivity in both liver and skeletal muscle [26]. This study illustrated that CD11c(+) macrophage populations (M1) are responsible for insulin resistance in obese animals and demonstrated that their continued presence is required to maintain this state. 

ATM M1 polarization status is not necessarily permanent; it can revert to M2 predominance under certain circumstances. For instance, switching mice from a HFD to a chow diet [27] or treating obese mice with thiazolidinediones (TZDs) [28] changed ATM polarization from M1 to M2 and subsequently improved insulin sensitivity. Although switching HFD to chow feeding reversed adipose tissue inflammatory cytokine levels, it did not change the quantity of CD11c(+) cells until 5 weeks after the diet change, indicating that the macrophage phenotype may be dynamic [27]. Furthermore, Shaul et al. demonstrated that not all CD11c(+) cells reflect the typical M1 phenotype as defined *in vitro* and in conditions of acute inflammation [29]. However, to summarize current understanding of a complex and emerging paradigm of adipose inflammation, M2 predominant ATMs are typically seen in normal, lean subjects while a transformation to the M1 state propagates the inflammatory state associated with obesity. 

## 6. Adipocyte Death and Crown-Like Structures 

Macrophage accumulation in VAT occurs in the context of continuous tissue remodeling that is pathologically accelerated in the obese state. Adipocytes increase and decrease in size in order to accommodate changes in lipid load during minor fluctuations in weight. With excessive weight gain, extreme increases in adipocyte size are accompanied by an elevated frequency of adipocyte death and macrophage accumulation [30]. The accelerated adipocyte death rate could partly be explained by hypoperfusion causing an inadequate supply of oxygen in the face of expanding adipose tissue. This phenomenon of poorly oxygenated adipose tissue was first observed in mice but has also been shown in obese humans [31]. Hypoxia activates the transcription factor hypoxia-inducible factor-1*α* (HIF-1*α*), which induces expression of various target genes; deletion of HIF-1*α* in adipocytes partially protects mice from HFD-induced obesity and insulin resistance compared with similarly fed wild-type controls [32, 33]. Strissel et al. tracked the adipocyte death rate in obese, HFD mice by assessing VAT histology periodically for 20 weeks and found that the proportion of dead adipocytes increased from <1% of total cells to >20% during the course of the study [30]. The adipocyte death rate was associated with parallel increases in weight, numbers of ATMs (expressing CD11c), TNF*α* and MCP-1 levels, and insulin resistance [30].

Macrophages are not homogeneously distributed throughout VAT but rather aggregated around dead adipocytes as shown in Figure 1. Dead adipocytes lack unilocular lipid droplets and therefore do not stain for perilipin [34]. Clusters of F4/80-staining macrophages surrounding perilipin (−) adipocytes are termed “crown-like structures” (CLS) [35]. Individual CLS contain up to 15 macrophages and the majority of ATMs are localized to CLS [35]. While it is rare to see CLS in chow-fed mice, there is greater than 10-fold increase in CLS number in HFD-fed mice [36]. In genetic and HFD models of rodent obesity, CLS are more numerous in visceral compared to subcutaneous depots, and CLS number correlates directly with insulin resistance [4, 35, 36]. The same holds true in obese humans, in whom CLS are significantly enriched in omental compared to inguinal adipose tissue [5]. Furthermore, the number of omental depot CLS correlates with local levels of inflammatory mediators, insulin resistance, and systemic vascular endothelial dysfunction, in humans [5, 37]. Taken together, CLS are pathological lesions in VAT of obese subjects, and they are highly correlated with adipose inflammation and insulin resistance. 

## 7. Inflammatory Signaling and Stress Responses Causing Insulin Resistance

**Figure 2:**
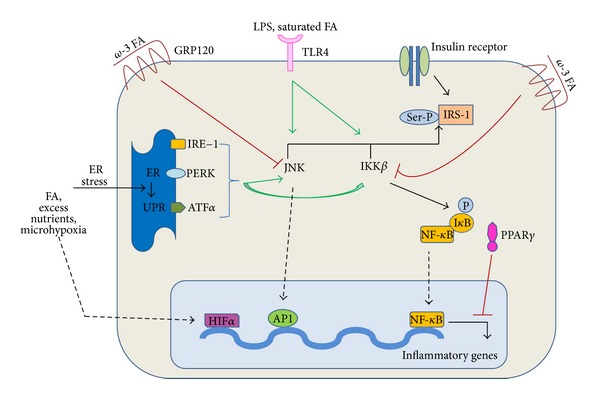
Various signaling pathways promoting or inhibiting inflammatory signaling (green arrows represent activation, and red arrows represent inhibition). Jun N-terminal kinase-1 (JNK1) and inhibitor of kB kinase (IKK*β*) are inflammatory signaling pathways which promote insulin resistance. Activation of either pathway leads to serine phosphorylation of insulin receptor substrate protein-1 (IRS-1), causing attenuation of insulin action. IKK*β* also phosphorylates inhibitor of nuclear factor-*κ*B (I*κ*B) which essentially frees nuclear factor-*κ*B (NF-*κ*B), allowing it to translocate to the nucleus, bind to DNA, and activate inflammatory mediators. JNK1 can also stimulate transcription of inflammatory genes in association with transcription factor activator protein 1 (AP1). Toll-like receptor 4 (TLR4) activation, which normally binds lipopolysaccharides (LPS) and saturated fatty acids (FA), results in activation of JNK and IKK*β*. Endoplasmic reticulum (ER) stress, stimulated by FA, nutrient excess, and microhypoxia, leads to the unfolded protein response (UPR). UPR comprises three main pathways: inositol-requiring enzyme (IRE)-1, protein kinase-like ER kinase (PERK), and activating transcription factor (ATF)*α* which all lead to activation of JNK1 and IKK*β*. Hypoxia also activates the transcription factor hypoxia-inducible factor-1*α* (HIF-1*α*), which induces expression of various target genes. Conversely, insulin sensitivity is promoted by activation of the omega-3 fatty acid receptor (GRP120) which inhibits JNK1 and IKK*β*. PPAR*γ* also promotes insulin sensitivity by interfering with the NF-*κ*B and AP1 signaling pathways and subsequent expression of inflammatory genes.

 Obesity-associated insulin resistance (IR) is consistently associated with elevated levels of proinflammatory cytokines such as TNF*α*, IL-6, and IL-1*β*, and neutralization of TNF*α* improves insulin sensitivity in obese rodents [38]. Both adipocytes and the M1 subset of ATMs are a major source of these cytokines. These cytokines activate inflammatory pathways that terminate in activation of Jun N-terminal kinase-1 (JNK1) and inhibitor of kB kinase (IKK*β*), the products of which alter signaling downstream of the insulin receptor and cause insulin resistance [39, 40]. Activation of these kinases in obesity highlights the intertwined relationship of metabolic and immune pathways; JNK and IKK are the same kinases that are activated in the innate immune response, mediated by Toll-like receptor (TLR) signaling stimulated by lipopolysaccharide (LPS), peptidoglycan, double-stranded RNA, and other microbial products (Figure 2).

### 7.1. IKK*β* Signaling Pathway

IKK*β* can impact insulin signaling through at least two pathways. First, it can directly phosphorylate insulin receptor substrate protein-1 (IRS-1) on serine residues, leading to attenuation of tyrosine kinase-mediated signaling from the insulin receptor, interference of normal insulin action, and subsequent insulin resistance [39]. Secondly, IKK*β* leads to phosphorylation of the inhibitor of nuclear factor-*κ*B (I*κ*B). In the resting unphosphorylated state, I*κ*B forms a complex with nuclear factor-*κ*B (NF-*κ*B), preventing it from entering the nucleus. However, phosphorylated I*κ*B dissociates from NF-*κ*B and undergoes degradation; free NF-*κ*B translocates to the nucleus, binds to DNA, and activates inflammatory mediators such as TNF*α* and IL-6 [41]. Arkan et al. showed that mice lacking IKK*β* in myeloid cells are insulin sensitive [42]. Moreover, when these myeloid-specific IKK*β* knockout mice were placed on HFDs, they became just as obese as their wild-type counterparts but were protected from obesity-induced glucose intolerance and hyperinsulinemia [42].

### 7.2. JNK1 Signaling Pathway

As seen in Figure 2, activation of JNK1 also results in inhibitory serine phosphorylation of IRS-1. In a manner similar to IKK*β*, JNK1 can stimulate transcription of inflammatory genes in association with transcription factor activator protein 1 (AP1) [43]. Knockout (KO) of JNK1 in nonhematopoietic cells protected mice from HFD-induced insulin resistance, in part through decreased adiposity [40, 44]. By contrast, mice with JNK1 knocked out of hematopoietic cells (macrophage-specific cells) became obese on HFD, with hepatic steatosis and increased intramuscular triglyceride content, but were still protected against insulin resistance [44]. Protection against insulin resistance was conferred to these hematopoietic cell-specific KO mice by a decrease in ATM content and reduction in inflammatory pathway gene expression [44]. This experiment demonstrates that obesity and tissue lipid burden may not be sufficient to cause insulin resistance. Without the inflammatory component, obesity does not lead to appreciably impaired insulin action as demonstrated in macrophage-specific IKK*β* and JNK1-KO mice [42, 44]. 

### 7.3. Toll-Like Receptors and Lipid Mediators

ATMs exist in a lipid-rich milieu, and free fatty acids, abundant in that milieu, can have a variety of effects on macrophage inflammatory pathways. For instance, omega-3 fatty acids are anti-inflammatory, polyunsaturated fatty acids are weak or neutral, and saturated fatty acids are proinflammatory [45]. Toll-like pattern recognition receptors recognize molecules that are broadly shared by pathogens. Specifically, Toll-like receptor 4 (TLR4) is expressed on macrophages and recognizes not only LPS produced by gram-negative bacteria but also saturated fatty acids; both of these ligands can activate TLR4, resulting in activation of JNK and IKK*β*. TLR4 expression is increased in obesity; when the gene encoding TLR4 was deleted, HFD mice were protected from insulin resistance and weight gain compared to controls [25, 46]. 

Lipid species appear to regulate inflammatory signaling in macrophages through non-TLR pathways as well. Overexpression of diacylglycerol transferase-1 (DGAT-1, which catalyzes the final stage in triglyceride synthesis) in macrophages protects against adipose macrophage infiltration, inflammation, and insulin resistance [47]. This suggests that the effects of triglycerides on macrophages may not be as inimical as the effects of the precursors of triglycerides, that is, fatty acids and diacylglycerols. Diacylglycerols in particular have been implicated in insulin resistance in liver and muscle [47]. In addition, ceramides have recently been shown to activate the nucleotide-binding domain, leucine-rich-containing family, and pyrin-domain-containing (NLRP) inflammasome [48]. NLRP activation ultimately leads to IL-1*β* and IL-18 secretion, a response that is generally stimulated by “danger signals” of nonmicrobial origin [48]. Finally, omega-3 fatty acids have anti-inflammatory and antidiabetic effects in humans and mice. Activation of the omega-3 fatty acid receptor (GRP120) on macrophages and adipocytes reverses adipose inflammation and insulin resistance in obese mice [49].

### 7.4. Endoplasmic Reticulum (ER) Stress

Multiple proinflammatory sources can lead to activation of ATMs in obesity, including ER stress. ER stress can be stimulated by fatty acids, nutrient excess, improperly folded proteins, and regional areas of microhypoxia, all of which occur in obese adipose tissue. As obesity develops, protein biosynthetic pathways are upregulated, activating the unfolded protein response (UPR) in the ER [50]. The UPR comprises three main pathways controlled by ER membrane proteins: inositol-requiring enzyme (IRE)-1, protein kinase-like ER kinase (PERK), and activating transcription factor (ATF)*α*. All 3 branches of the UPR can directly engage inflammatory pathways through activation of IKK*β* and/or JNK signaling [50]. In summary, there are numerous mechanisms propagating inflammation in obesity, but most if not all appear linked to one of two final common pathways—JNK and IKK*β*—which lead to the end-result of insulin resistance. 

## 8. Mechanisms to Counteract Inflammatory Signaling

### 8.1. Peroxisome Proliferator-Activated Receptors (PPARs)

PPAR*γ* is a member of the nuclear receptor superfamily of ligand-dependent transcription factors that is predominantly expressed in adipose tissue and the intestines. PPAR*γ* is also highly expressed in macrophages and is a natural ligand to FFAs and eicosanoids. PPAR*α* and PPAR*γ* function as regulators of lipid metabolism and glucose homeostasis, respectively, and are targets for fatty acid oxidizing fibrates and insulin-sensitizing TZDs, respectively [51]. As seen in Figure 2, PPAR activators inhibit the activation of inflammatory response genes by interfering with the NF-*κ*B and AP1 signaling pathways, thereby promoting insulin sensitivity [51]. Consistent with this effect, Hevener et al. demonstrated that macrophage-specific PPAR*γ* KO induced glucose intolerance with skeletal muscle and hepatic insulin resistance in lean mice fed a normal diet [52]. This phenotype was associated with increased expression of inflammatory markers and impaired insulin signaling in adipose tissue, muscle, and liver. Furthermore, insulin resistance became more severe in mice lacking macrophage PPAR*γ* following HFD feeding, and these mice were only partially responsive to TZD treatment [52]. Another member of the PPAR family, PPAR*β*/*δ*, is induced by IL-4 and IL-13 to promote alternative activation of macrophages. Myeloid-specific deletion of PPAR*β*/*δ* in mice has been shown to cause insulin resistance with increased adipocyte lipolysis and severe hepatosteatosis [53]. Thus PPARs function in an anti-inflammatory manner and promote M2 polarization.

## 9. Other Immune Cells in Adipose Tissue Contributing to Insulin Resistance

As obesity develops, enlarging adipocytes secrete chemokines that attract immune cells. Macrophages are amongst the earliest immune cells to infiltrate adipose tissue, as their numbers increase after one week of HFD [43]. Although macrophages are vital in innate and adaptive immunity, the immune response is a result of interactions between multiple cell types. Hence, regulatory T cells, CD8+ effector T cells, B cells, mast cells, and eosinophils within adipose tissue have also been implicated in the pathogenesis of obesity-related insulin resistance. 

### 9.1. Lymphocytes

T cells are involved in adipose tissue inflammation and IR by modifying ATM numbers and affecting polarization states. T-helper (T_H_) cells express the cell surface marker CD4 and comprise T_H_1 cells (which produce proinflammatory cytokines) and T_H_2 cells (which produce anti-inflammatory cytokines). Regulatory T cells (Tregs), another CD4(+) type, secrete anti-inflammatory signals, inhibit macrophage migration, and induce M2 polarization [54]. Feuerer et al. discovered that Tregs were highly enriched in the VAT of normal mice, but their numbers were strikingly reduced at this site in HFD-fed and genetically obese mice [54]. Furthermore, Treg deletion in mice induced acute elevations of TNF*α* and IL-6 transcripts in VAT, while inhibiting insulin signaling in muscle and liver [54]. In contrast to CD4(+) cells, T cells that express the surface antigen CD8 (known as effector or cytotoxic T cells) promote ATM accumulation and proinflammatory gene expression and are increased in number in obese adipose tissue [55]. Winer et al. highlighted the role of Tregs through analyses of T-cell deficient Rag-1(−/−) mice [56]. When placed on HFD, Rag-1(−/−) rodents were more insulin resistant than their controls. Adoptive transfer of CD4(+) to these rodents reversed the insulin resistant phenotype and abrogated VWAT inflammation; this was not observed with adoptive transfer of CD8(+) cells in Rag-1(−/−) rodents [56]. A critical unanswered question in regard to these profound effects of T cells is what in the milieu of adipose tissue activates them in the first place. It would be important to determine if the activator is a specific antigen or whether some form of completely antigen-independent T cell activation takes place.

B cells have also been ascribed functions in promoting VAT inflammation during weight gain. Winer et al. identified a VAT B-cell population that expanded with HFD feeding, with parallel increases in tissue IgM and IgG levels. Immunohistochemistry revealed deposits of these antibodies throughout VAT that mirrored the locations of CLS [57]. Mice lacking the immunoglobulin mu heavy chain (B-null mice) were protected from HFD-induced insulin resistance and displayed diminished VAT inflammation as indicated by lower numbers of M1-like macrophages and activated CD8(+) T cells. Strikingly, reconstitution of B-null mice with either wild-type (WT) B cells or serum immunoglobulins from WT HFD-fed mice was sufficient to restore the insulin resistant phenotype. Furthermore, adoptive B-cell transfer to lean WT mice induced insulin resistance [57]. These results demonstrate inflammatory pathways in which B cells and adaptive immunity play a role in insulin resistance. Again, the stimulus for B-cell activation, whether a specific antigen or otherwise, remains to be specified.

### 9.2. Mast Cells

Although mast cells are well known for their role in allergy and anaphylaxis, they seem to play a role in obesity as well. Altintas et al. found that subcutaneous fat of lean mice contained more mast cells, but fewer solitary macrophages and CLS than visceral fat [4]. With obesity, there was no significant change in mast cell density of subcutaneous fat, but there was a substantial increase in mast cell number in visceral fat. CLS became prevalent in visceral fat of obese mice, and their distribution paralleled that of mast cells. Immunofluorescence staining and confocal microscopy demonstrated that a subset of mast cells in adipose tissue contained and released preformed TNF-*α* as well [4]. In summary, subcutaneous fat differed from visceral fat by not only immune cell composition but also by having a lower prevalence of CLS both in lean and obese mice. The increase in mast cells in visceral fat of obese mice suggests a role in the pathogenesis of obesity and insulin resistance. Liu et al. discovered that in mice fed on a Western diet, genetically induced deficiency of mast cells or their pharmacological stabilization (with daily injections of disodium cromoglycate) reduces body weight gain and levels of inflammatory cytokines in serum and VAT, in conjunction with improved glucose homeostasis and energy expenditure [58].

### 9.3. Eosinophils

Similar to mast cells, eosinophils mediate allergic reactions in addition to combating parasites. Wu et al. demonstrated that eosinophils also participate in endorsing a M2-like ATM polarization state via IL-4 [59]. By analyzing VAT of mice on a normal chow diet, these investigators found that >90% of the IL-4-competent cells recovered were eosinophils. There was a reciprocal relationship between adipose eosinophil quantity and mouse weight. When placed on a HFD, those mice that were genetically deficient in eosinophils had increased body fat, impaired glucose tolerance, and insulin resistance in comparison to WT controls. Wu et al. also observed that mice on a HFD who were infected with helminths sustained a metabolic response characterized by decreased fasting glucose and improved insulin sensitivity from the early postinfection phase and sustained up to 35 days following infection [59].

## 10. Conclusion

 At a histological level, adipose tissue inflammation in obesity is associated with macrophage accumulation and development of CLS. At a cellular level, the resultant insulin resistance can be explained by activation of JNK1 and IKK*β*, two critical pathways mediating a range of inflammatory and stress mechanisms activated in obesity. It is interesting to note that many cellular and biochemical components of the immune system that normally protect the host from foreign pathogens, such as macrophages and TLRs, also play a pathologic role in obesity-related inflammation. Overall, adipose tissue inflammation in obesity demonstrates that the immune system and metabolism are highly integrated.
